# Exceptionally preserved asphaltic coprolites expand the spatiotemporal range of a North American paleoecological proxy

**DOI:** 10.1038/s41598-020-61996-y

**Published:** 2020-03-19

**Authors:** Alexis M. Mychajliw, Karin A. Rice, Laura R. Tewksbury, John R. Southon, Emily L. Lindsey

**Affiliations:** 1 La Brea Tar Pits & Museum, 5801 Wilshire Blvd, Los Angeles, California 90036 USA; 2 Institute of Low Temperature Science, Hokkaido University, Sapporo, 060-0819 Japan; 3 Laboratories of Molecular Anthropology and Microbiome Research, University of Oklahoma, Norman, OK 73019 USA; 4 Department of Earth System Science, UC Irvine, Keck CCAMS Group, Irvine, California 92697 USA

**Keywords:** Palaeoecology, Stable isotope analysis, Zoology

## Abstract

As fossilized feces, coprolites represent direct evidence of animal behavior captured in the fossil record. They encapsulate past ecological interactions between a consumer and its prey and, when they contain plant material, can also guide paleoenvironmental reconstructions. Here we describe the first coprolites from the lagerstätte Rancho La Brea (RLB) in Los Angeles, California, which also represent the first confirmed coprolites from an asphaltic (“tar pit”) context globally. Combining multiple lines of evidence, including radiocarbon dating, body size reconstructions, stable isotope analysis, scanning electron microscopy, and sediment analyses, we document hundreds of rodent coprolites found in association with plant material, and tentatively assign them to the woodrat genus *Neotoma*. *Neotoma* nests (i.e., middens) and their associated coprolites inform paleoclimatic reconstructions for the arid southwestern US but are not typically preserved in coastal areas due to environmental and physiological characteristics. The serendipitous activity of an asphalt seep preserved coprolites and their original cellulosic material for 50,000 years at RLB, yielding a snapshot of coastal California during Marine Isotope Stage 3. This discovery augments the proxies available at an already critical fossil locality and highlights the potential for more comprehensive paleoenvironmental analyses at other asphaltic localities globally.

## Introduction

Coprolites are some of the most important ichnofossils that can be recovered from a diversity of taphonomic, ecological, and geologic contexts^1^. As trace fossils, coprolites represent windows into the evolution of ecological interactions such as predation, herbivory, and parasitism, and can contain paleoecological proxies spanning thousands to millions of years in the past^2–4^. In deep time, they are permineralized or lithified, constituting a cast or mold of the original fecal matter^5^. Conversely, coprolites containing their original biological material have been recovered from a taxonomically diverse range of Quaternary vertebrates including, for example, ground sloths^6^, mammoths^7^, crocodiles^8^, and moas^9^. The intact digested matter present in Quaternary coprolites can provide crucial evidence for testing ecological questions that may be otherwise difficult to address in the fossil record and that can be examined with techniques suitable for bridging fossil and modern samples (e.g., identification of plant macrofossils and pollen, ancient DNA, and biomarkers^10^). Despite these advantages, the preservation of Quaternary coprolites is biased to cold or arid climates, permanently wet sites, caves, rock shelters, and/or high elevation areas, thereby excluding many ecosystems, deposits, and taxonomic groups from this form of study.

Rancho La Brea (RLB) is a famous Quaternary lagerstätte that has yielded >5 million specimens and is located in Los Angeles, California (34°03′48″N, 118°21′20″W). Studies leveraging RLB’s abundant and well-preserved vertebrate remains have defined our understanding of late Pleistocene carnivores, through studies of morphological change^11^, pathologies^12^, and trophic interactions^13,14^. Far less attention, however, has historically been focused on primary producers and the lower trophic levels of food webs, thus hindering the current use of the site for paleoenvironmental proxies despite its potential (though see recent entomological progress^15^). While known colloquially as the “La Brea Tar Pits”, the site actually comprises a series of asphaltic deposits containing fossil material in a matrix of gravels, sands, silts, and clays—the “pit” shapes are the result of human excavations that subsequently refilled with liquid asphalt^16^. RLB sits above the Salt Lake Oilfield, and throughout the Late Quaternary, Miocene-aged hydrocarbons migrated upwards from the Puente Formation reservoir (Monterey Formation equivalent in the Los Angeles Basin^17^), resulting in periodically surficial liquid asphalt that infiltrated sediments, entrapped organisms, and preserved biological tissues including bone collagen, insect chitin, plant cellulose, and mollusk shells^18^. While RLB’s climatic and elevational qualities do not align with optimal coprolite recovery conditions given its low elevation, non-arid coastal setting that favors decomposition rather than desiccation, the biological material of coprolites could theoretically be preserved by rapid impregnation with liquid asphalt.

The vast majority of catalogued RLB specimens originated from >90 deposits (“pits”) that were excavated with a near-exclusive focus on the bones of large mammals and birds in the early 1900s^18^. Reconstructions based on megafaunal remains consider the site to have operated as a “trap” in which carnivores were attracted to struggling herbivores stuck in pooling surficial asphalt, subsequently died, and were rapidly covered^19,20^. Extensive disarticulation of faunal elements and lack of stratigraphic integrity in RLB deposits have been cited to invoke churning of viscous, asphalt-saturated sediments and/or trampling by panicked animals as primary processes in the formation of RLB fossil deposits^19,20^, both of which could preclude the preservation of intact coprolites. Other models that incorporate geologic lines of evidence instead emphasize the roles of fluviatile agents and burial by alluvium from the Santa Monica Mountains; such a high-energy depositional environment could also be unfavorable for coprolite preservation^16^.

**Figure 1:**
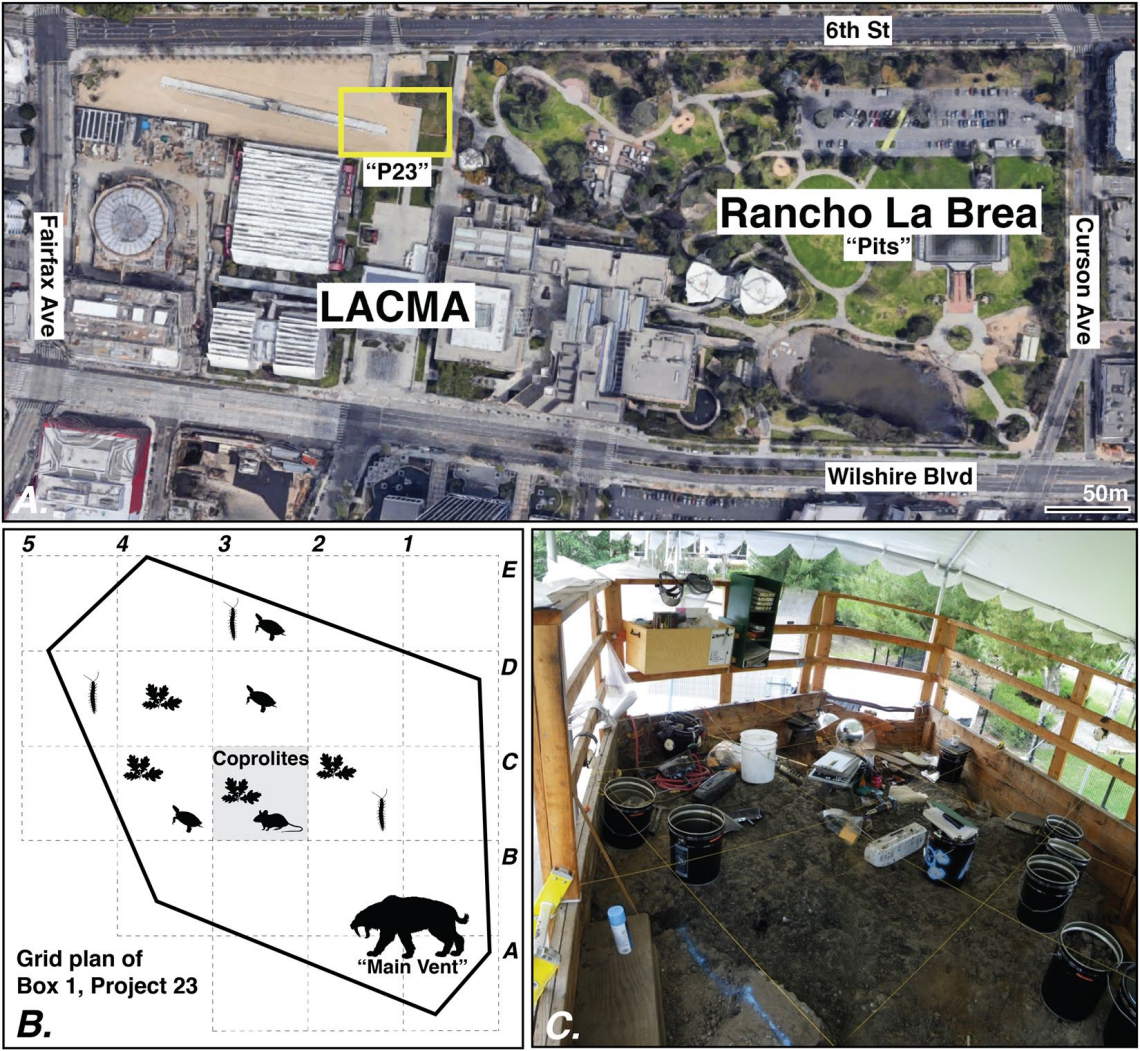
(**A**) Map of Hancock Park in Los Angeles, California, highlighting the area of historic “pit” excavations of Rancho La Brea in relation to Project 23 “P23” deposits (yellow rectangle). Construction of an underground parking structure at the Los Angeles County Museum of Art (LACMA) – adjacent to what is today the La Brea Tar Pits & Museum – revealed 16 asphaltic deposits that were placed into 23 protective boxes (including the coprolite yielding Box 1). Map imagery © Google, Maxar Technologies, US Geological Survey, and the USDA Farm Service Agency. (**B**) Diagram of Box 1 showing the 2-dimensional grid labeling system used in Box 1, where each unit is assigned an alphanumeric code (A–E, 1–5; e.g, “C3”). The saber-tooth cat symbol indicates the location of the megafauna-rich “main vent” (grids A1–2 and B1–2); oak leaf symbols indicate the presence of plant mats; millipede symbols indicate the presence of articulated millipedes; turtle symbols indicate the presence of intact turtle shells. The rodent symbol indicates where the coprolites described in this study were discovered in the gray highlighted cell of C3. Plant and animal symbols modified from PhyloPic. (**C**) Example of excavation processes and use of grid system within the southern portion of Box 1 (near A and B units).

A new opportunity to systematically excavate RLB deposits arose when, in 2006, the construction of an adjacent underground parking garage for the Los Angeles County Museum of Art (LACMA; Fig. 1A) unearthed 16 new fossiliferous asphaltic deposits. Entire blocks were exposed and pedestalled, and custom boxes were then built around deposits, allowing them to be removed and transplanted intact from their *in situ* position in 23 custom-built wooden boxes (“Project 23”). Project 23’s Box 1, weighing ~123,000 lbs and measuring 4 m wide x 5 m long x 2 m deep, was the first to be excavated, and has yielded >25,000 specimens^21,22^. Excavation efforts in Box 1 were focused primarily on the main vertebrate deposit of coarse sediment (referred to as the “main vent” for consistency with prior work^22^; grids A1–2/B1–2; Fig. 1B) that most closely mirrored a “typical” fossiliferous RLB deposit containing the remains of ungulates such as *Bison, Camelops, Odocoileus* and *Equus;* the carnivores *Canis, Lynx, Panthera, Puma*, and *Smilodon*; and the ground sloths *Nothrotheriops* and *Paramylodon*^22^.

Here we report the first confirmed coprolites in the 100-year history of RLB’s extensive exploration and the first confirmed asphalt-preserved coprolites, providing new taphonomic scenarios for both RLB and the known preservation conditions of coprolites. We combine multiple lines of evidence to identify and describe the coprolites and their paleoecological significance, including radiocarbon dating, stable isotope analysis, scanning electron microscopy (SEM), and comparative measurements. Both body size reconstructions and a plant macrofossil context suggest that the coprolite producer was of the genus *Neotoma*, a group of rodents whose middens form the backbone of paleoclimatic inferences across arid and high elevation ecosystems of North America. These specimens provide a direct window into the behavior of a still-extant small mammal and the base of a southern California food web ~50,000 years ago, during Marine Isotope Stage 3 prior to the last glacial maximum. As there are three other fossiliferous asphaltic localities in California alone (Carpinteria, McKittrick, and Maricopa^18^) and more than a dozen globally across the Americas and Eurasia^23^, we suggest that closer inspection using quantitative analyses for identifying taphonomic diversity would help improve palaeoecological reconstructions based on these deposits.

## Results

### Fecal pellet discovery in Box 1

Rodent fecal pellets were first noted in matrix washing records by excavators in 2016 while following standard Project 23 salvage processing protocols, which include asphalt removal with n-propyl bromide and bulk sieving^24^. The fecal pellets were first thought to be modern contamination produced by the invasive rats prevalent in urban Los Angeles (black rat, *Rattus rattus*, or brown rat, *Rattus norvegicus*). The pellet-containing grids in Box 1 were found outside of the megafauna-containing “main vent” and included articulated millipedes, birds, turtles, and plant mats in the context of finer sands, silts, and clays. The fecal pellets in this study were found in the grid square C3 and excavation depth level 7 (thus the unit C3/L7, Fig. 1B), that differed from typical coarse-grained RLB asphaltic disarticulated vertebrate deposits^25^. Subsequent analyses of bulk sediment in 2018 demonstrated that material from the unit C3/L7 contained higher than average percent asphalt content as compared with “typical” RLB sediments (~35–45% asphalt for C3/L7 as compared with ~5–10% asphalt for Box 13, B3/L5^25^).

### Determination of fecal pellet age

Hydrocarbon contaminants such as asphaltenes were removed from the pellets to produce radiocarbon dates on bleached holocellulose^22^. Direct radiocarbon dates on two individual pellets yielded ^14^C ages of >47,000 years before present (ybp), confirming that these fecal pellets are indeed coprolites and are not the result of modern contamination by invasive *Rattus* in a highly urbanized area (Table 1). While the probability distributions of all of the dates extend well beyond the range of the IntCal13 calibration^26^, the overall trend of that curve suggests median calibrated ages close to 50,000 ybp or older, with likely calibrated uncertainties of 1000–2,500 ybp. The two coprolite dates (UCIAMS-199130, UCIAMS-203246) overlap with their large errors. Therefore, it is reasonable to suggest that the coprolites were either deposited contemporaneously by one or multiple individuals or by a series of individuals occupying a transgenerational midden, as is the typical scenario for paleomiddens across North America^27^. We also produced two dates on oak leaves accompanying the coprolites (UCIAMS-220767, UCIAMS-220768), confirming the stratigraphic association of the material within the unit C3/L7. This temporal range is ~10,000 years older on average than the more than 30 radiocarbon dates previously produced on fossils excavated from Box 1’s “main vent”^21,22^.

**Table 1:** Uncalibrated radiocarbon dates produced on coprolites and their associated oak (*Quercus*) material from Box 1 unit C3/L7.

UCIAMS #	Specimen	^14^C age	±	δ^13^C	Mass of dated C
199130	Coprolite (P23–33721a)	46900	1500	−24.7	0.77 mg
203246	Coprolite (P23–33721b)	49000	2600	−25.5	0.73 mg
220767	Oak Leaves (P23–39548)	46140	810	na	0.78 mg
220768	Oak Leaves (P23–39539)	47600	1300	−24.4	0.41 mg

### Identity of the coprolites

**Figure 2:**
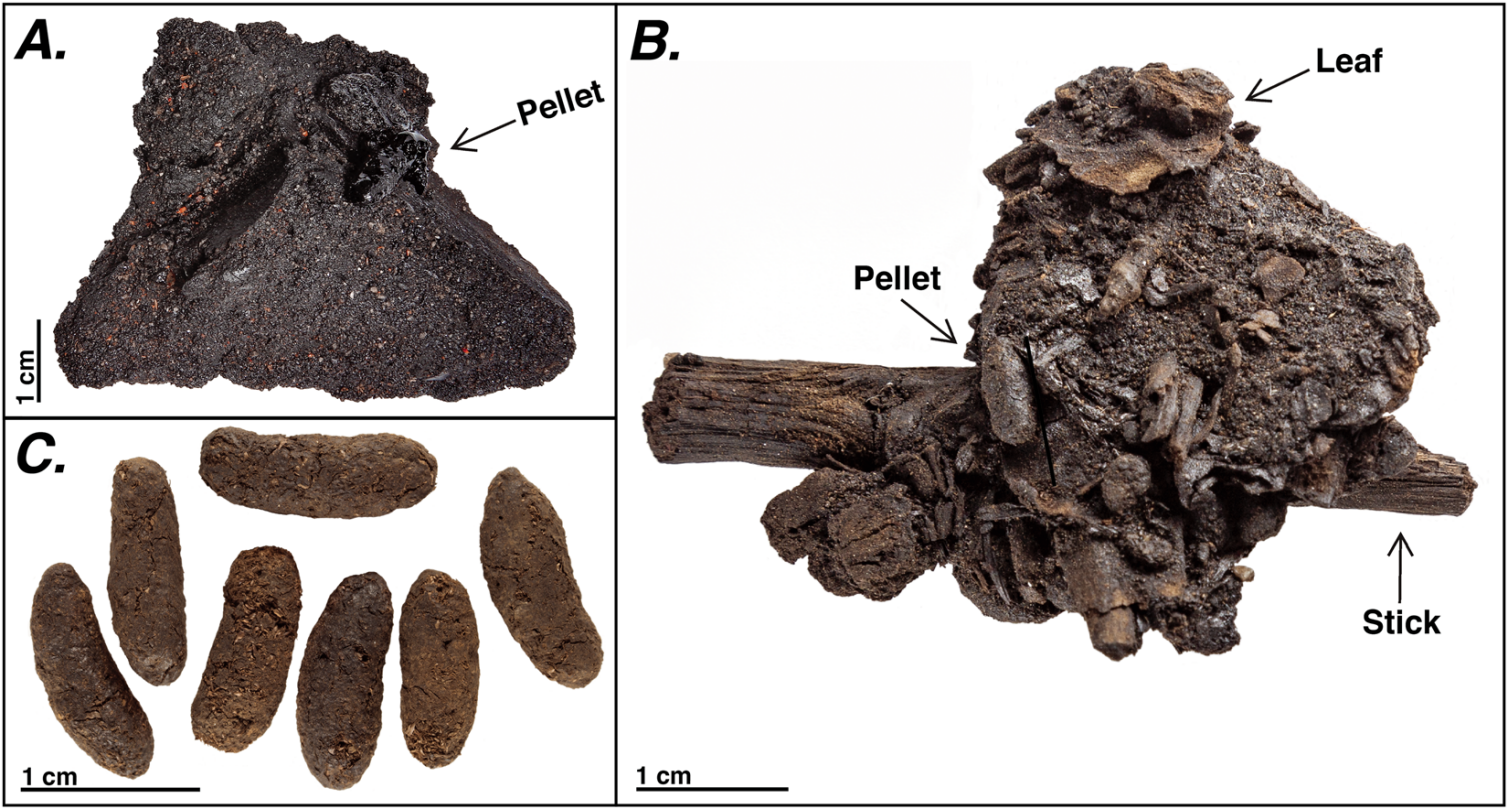
Example photographs of Rancho La Brea coprolites (**A**) *in situ* example of bulk matrix from Project 23, Deposit 1, Grid C3/L7 prior to cleaning; visible coprolite indicated by arrow (P23–39536 - GUID e66a891d-c330-4de9-8448-a5f2cb8f55de); (**B**) *in situ* example of cleaned but non-disaggregated bulk matrix from grid C3/L7 featuring visible coprolites and plant material (P23-37336 - GUID b0844069-0902-4f5e-b650-97fe152d9f7d); (**C**) isolated coprolites sieved from cleaned matrix (batch P23-33721- GUID 074a519d-024f-436a-b5af-9b17073f84d6). Photographs courtesy of Carrie Howard.

**Figure 3:**
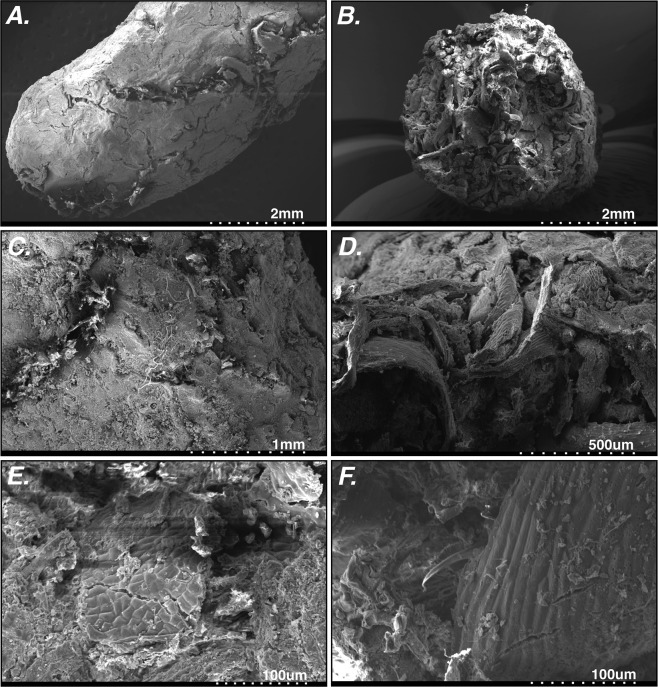
SEM images of coprolite surface and interiors, including visible plant fibers and materials. Scale bar depicted on each individual image. Coprolites featured: (**A**) P23-33821; (**B**) P23–38819; (**C**) P23–33820; (**D**) P23–38819; (**E**) P23–33820; (**F**) P23–38819.

The coprolites are cylindrical pellets that represent terrestrial mammal feces (“group III” characterization^28^) found in a context of plant macrofossils, fine sediments, and a high percent asphalt content (Fig. 2). SEM imaging revealed the presence of plant material within the coprolites, consistent with an herbivorous rodent as the producer (Fig. 3). This was corroborated by average carbon stable isotope (δ^13^C) values of −24.5 ± 0.59 indicative of a diet comprising C3 plants, and a mean %C of 43.2% **±** 1.50 on cellulosic material (n = 10) (Supplemental Material). A number of small mammal skeletal elements have been recovered from other grid/level units within Box 1, spanning four orders including Lagomorphs, Eulipotyphlans, Chiropterans, and Rodents. Rabbits (Lagomorpha: Leporidae: *Lepus californicus*, *Sylvilagus audubonii*, *Sylvilagus bachmani*) can be eliminated based on pellet size and the cylindrical (as opposed to rounded) morphology. An exclusively herbivorous diet is also inconsistent with the resource use of eulipotyphlans such as shrews (e.g, *Sorex ornatus*) as well as insectivorous rodents (e.g., *Onychomys* sp.). Potential rodent candidates that can be discerned by coprolite shape, size, and consistency include geomyids (*Thomomys bottae*), heteromyids (*Perognathus*, *Dipodomys*), sciuirids (*Otospermophilus*, *Eutamias*), and cricetids (*Microtus*, *Peromyscus*, *Reithrodontomys*, and *Neotoma*).

The sheer quantity of pellets (hundreds, potentially thousands) and location in an exceptionally large mass of plant macrofossils is consistent with a rodent nest, such as the middens produced by woodrats (*Neotoma* sp.). The surface of each pellet is generally smooth with small fissures that expose what appear to be plant fibers (Fig. 3). A measured sample of 431 randomly selected cylindrical pellets from the unit C3/L7 had an average length of 9.59 ± 1.34 mm, ranging from 6.25–13.37 mm, and a width of 3.86 ± 0.47 mm, ranging from 2.43–5.43 mm. Such measurements are consistent with the widths of fecal pellets reported in the literature for the genus *Neotoma* (Table 2). Pellet width is more consistently diagnostic of rodent species as diet items can generate variable pellet lengths^29^.

**Table 2:** Average coprolite widths from Rancho La Brea in comparison with modern *Neotoma fuscipes* and *Neotoma lepida* fecal pellet widths and standard deviations, and a Late Pleistocene *Neotoma lepida* coprolite sample from the literature.

Species	Locality in California	Width	SD	N	Age
Coprolites	Rancho La Brea	3.86	0.47	431	Late Pleistocene
Coprolites	Rancho La Brea	4.23	0.26	215 (Widest 50%)	Late Pleistocene
Coprolites	Rancho La Brea	4.60	0.20	Widest 50	Late Pleistocene
Coprolites	Rancho La Brea	4.76	0.24	Widest 20	Late Pleistocene
*N. fuscipes*	Wildcat Canyon, Berkeley^35^	4.2	0.85	96	Modern
*N. lepida*	Titus Canyon, Death Valley^71^	4.79	0.12	Widest 20	Modern
*N. lepida*	Titus Canyon, Death Valley^71^	4.87	0.08	Widest 20	Modern
*N. lepida*	Titus Canyon, Death Valley^71^	4.88	0.1	Widest 20	Modern
*N. lepida*	Titus Canyon, Death Valley^71^	4.84	0.11	Widest 20	Late Pleistocene

**Figure 4:**
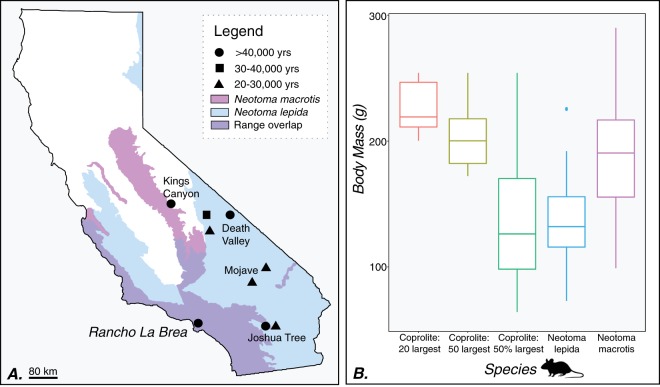
(**A**) Extant ranges of *Neotoma macrotis* and *Neotoma lepida* from the California Department of Fish and Wildlife's Life History and Range database (https://www.wildlife.ca.gov/Data/CWHR/Life-History-and-Range). Symbols depict locations of pre-Last Glacial Maximum general occurence localities of coprolite-containing *Neotoma* middens, taken from the USGS/NOAA North American Packrat Midden Database (version 4, June 2016, https://geochange.er.usgs.gov/midden/search.html). The map was produced using ArcGIS Online (ESRI), R, and Adobe Illustrator. (**B**) Comparison of estimated body size for Rancho La Brea *Neotoma* with weight at capture for *Neotoma lepida* (N = 59) and *Neotoma macrotis* (N = 30) specimens collected from Los Angeles county, accessioned at the Natural History Museum of Los Angeles County Mammalogy collections (LACM). Three outlier large coprolites were excluded for visualization— see supplemental data file for details. Rodent symbol modified from PhyloPic.

As is consistent with the *Neotoma* literature, we assume that these fecal pellets represent multiple generations of individuals and therefore are a population-level estimate of fecal pellets, rather than representing fecal pellets produced from a single individual rodent; intergenerational use of one midden is the standard assumption of paleontological studies and has been documented in the modern day^27^. We estimated body size using an equation that relates body mass to width of the fecal pellets^29^. Various body size estimates that account for ontogeny and exclusion of juveniles/subadults, including the widest 50%, widest 50, and widest 20 pellets, are consistent with the genus *Neotoma*, in particular the two species that are known to occupy Los Angeles county in the present day: *Neotoma macrotis* (the big-eared woodrat) and *Neotoma lepida* (the desert woodrat) (Fig. 4A). While we use the species names *macrotis* and *lepida* to remain consistent with local museum collections, we recognize there have been recent systematic revisions for the *Neotoma lepida* group^30^ that are not reflected in museum labels (e.g, skeletal remains at RLB are listed as *Neotoma fuscipes* but *Neotoma fuscipes macrotis* has been elevated to *Neotoma macrotis*^31^); we are primarily interested in an identification at the genus level. Coprolite-based estimates of body size range from ~100–200 grams, which is also consistent with measurements of museum specimens of *Neotoma* collected in Los Angeles county (Fig. 4B). Such estimates may represent a conservative underestimate of body mass given the effects of pellet desiccation and asphalt removal.

## Discussion

Coprolites provide one of the most direct windows into past behaviors and ecological interactions available in the paleontological record. *Neotoma* middens and the coprolites and plant macrofossils contained within them have come to define our understanding of Quaternary vegetation in the arid southwestern US^32^. While the USGS-NOAA midden database contains ~3,200 individual middens (USGS/NOAA North American Packrat Midden Database v.4; https://geochange.er.usgs.gov/midden/search.html), nearly all of these are restricted to the arid southwest due to favorable environmental preservation factors and physiological variations including the viscous urine of desert dwelling species adapted to dry conditions that hardens into a resinous substance called amberat^33^. Recent studies have highlighted the limitations of this ecological and spatial restriction to such preservation conditions, and the exclusion coastal regions in forming a broader narrative of North American vegetation change^34^. The RLB material described here represents the first occurrence of *Neotoma* coprolites and associated plant material from the Pacific coast (Los Angeles) and are therefore a new geographic contribution to the paleoecological proxy literature.

Their size, physical appearance, isotopic composition, and depositional context within significant quantities of plant macrofossils suggest that the fecal pellets from RLB represent coprolites produced by rodents of the woodrat genus *Neotoma*. *Neotoma* droppings typically accumulate within the nest chamber of a midden and each individual rodent can produce enormous quantities of fecal pellets; for example, a single captive female has been documented to produce 144 pellets over the course of just 24 hours^35^. This occurrence represents the westernmost locality for *Neotoma* coprolites, distinctive in both its present-day low elevation (~50 masl) and proximity to the Pacific coastline. The RLB coprolites are also exceptional in their age of ~50,000 ybp, within Marine Isotope Stage 3 (MIS3)^36^ (Table 1); of the 345 midden occurrences in California, less than 1% pre-date 40,000 ybp, and <2% recorded occurrences are older than 30,000 ybp. Moreover, all of the pre-30,000 ybp middens in California represent localities characterized by high elevations and/or arid climates (e.g, Kings Canyon, Joshua Tree, Death Valley, Mojave Desert^37–41^); Fig. 4).

Two woodrat species co-occur in the Los Angeles region today: the big-eared woodrat, *Neotoma macrotis*, and the desert woodrat, *Neotoma lepida* (Fig. 4). Today, *Neotoma lepida* is typically associated with arid environments and is common in pinyon-juniper, sagebrush, and desert habitats including Joshua Tree and the Mojave Desert^42^. *Neotoma macrotis* prefers chaparral with moderate tree canopy and understory brush and is a specialist on oak (*Quercus agrifolia*) in the western coastal Santa Ana mountains of California^43^. It is possible that *Neotoma fuscipes* was present during the cooler temperatures of the Late Pleistocene and its range has shifted northwards in the Holocene and present day. However, *Neotoma fuscipes* and *macrotis* form hybrid zones today in central California^44^ and as distinguishing the evolutionary history of these two species is difficult in the present-day, we do not attempt to do so from coprolites alone. Ancient DNA analyses have so far been unsuccessful for RLB vertebrate material^45^, precluding the use of genetic identification techniques for *Neotoma* species in RLB deposits for now.

The range of RLB coprolite-based body size estimates, and thus species identifications, depends on the number of coprolites considered as the representative population. Using the largest fecal pellets for a population estimate^29^, the widest 20–50 coprolites would be consistent with *Neotoma macrotis*, yet the larger sample (top 50% of all pellets) encompasses variation that extends to *Neotoma lepida*. Members of the genus *Neotoma* have been shown to follow Bergman’s rule, where populations experiencing colder temperatures exhibit larger average body sizes^46^. Therefore, this size variation could represent either within-species responses to past temperature fluctuations captured in the intergenerational record or could be due to species turnover at the site in response to changing microclimates or vegetation at the local scale. Including the full error range and calibration uncertainty, the temporal span of the RLB coprolite radiocarbon dates stretches across two Greenland interstadials (numbers 12, 13) and an intervening stadial during MIS3^47^. Regional climate records from lake cores suggest a more mesic and cooler MIS3^48,49^. However, these records are from higher elevation areas; for example, today Lake Elsinore is 400 meters above sea level, while RLB is just 50 meters above sea level and therefore Lake Elsinore may not reflect the local conditions of the Los Angeles basin. The only available quantitative paleoclimate record for the Los Angeles basin is derived from insect-based temperature analogues from RLB, which suggest local conditions were more aligned with modern values, and less mesic and cool than suggested by lake cores (mean summer temperatures within ±5 °C of modern conditions and summer precipitation from 0–40mm^15^). Unfortunately, paleoclimate inferences from RLB’s existing botanical descriptions are currently considered unreliable due to the lack of stratigraphic and taphonomic context (including allochthonous vs. autochthonous origins) for curated isolated specimens^50,51^. There is thus a clear need to develop additional quantitative microclimate and vegetation proxies at RLB, such as the plant macrofossils associated with our stratified C3L7 unit, which are of direct relevance to furthering paleoecological interpretations of vertebrates^13,22^.

As a lagerstätte, RLB is well-known for both its preservation of an exceptional abundance of specimens as well as a diversity of taxonomic groups and tissues including collagen, chitin, and cellulose^18^. The only published reports of ichnofossils at RLB are from insects: post-mortem bone damage resulting from carrion-consuming beetles^52^ and intact wasp galls^53^. Insects also represent the only record of fossilized nest building behavior documented at RLB: a specimen of fossilized leafcutter bee (*Megachile gentilis*, LACMRLP 388E from Pit 91) preserves pupal morphology and nest cell construction in three dimensions^54^. For such preservation to occur, the leafcutter bee likely created its nest first in a non-asphaltic area, and this nest was subsequently infiltrated by a newly active asphalt seep— a probable scenario for the coprolite-associated plant material reported here.

Vertebrate behavior has been inferred at RLB indirectly via the study of pathologies^12^, morphological proxies (e.g, dental microwear^55^), and stable isotopes of bone collagen^13,22^ and tooth enamel^14^. The coprolites, as the first fossilized diet product at RLB, are thus unique in directly capturing a record of vertebrate behavior: in this case, *Neotoma*’s consumption of leaves and other plant fibers, as evidenced in SEM images of coprolite cross-sections. Plant tissues vary distinctively in the ranges of their percent carbon (%C) values: leaves are 42–50% C, stems are 46–48% C, and wood/twigs are 40–44% C^56^. With an average %C of 43.2 ± 1.50%, the RLB coprolites are consistent with a *Neotoma* diet of leaves and/or twigs.

Stable isotopes of carbon (δ^13^C) are a widely used tool in evaluating the diets and food web linkages of past organisms as it relates to primary producer pathways (e.g., C3 vs. C4 plants). Previous research at RLB has pointed to largely C3 vegetation conditions in the immediate area based on the δ^13^C values of megafauna bone collagen^13,22^. The *Neotoma* coprolites have a mean δ^13^C of −24.5‰ ± 0.59, suggesting the meals consisted of C3 plants and that the rodents foraged in a closed wooded environment. Although plant stable isotopes have seldom been studied at RLB, δ^13^C values of a contemporaneous C3 plant, juniper (*Juniperus*) from 40,140–68,000 years before present have been reported as −19 to −22‰^57^ and a single unidentified piece of “wood” has yielded a δ^13^C value of −20.2‰ from Project 23’s Box 1^22^. Correcting for the enrichment between diet item and consumer (a rodent specific feces-diet fractionation of δ^13^C_feces-diet_ = Δ = −3.77‰^58^), the RLB *Neotoma* δ^13^C values could be more consistent with consumption of juniper, *Juniperus sp*., rather than oak (estimated diet value δ^13^C = −20.73‰; RLB juniper values −19–22‰; P23–39539 oak = −24.4‰). Modern *Neotoma* are known to consume juniper when it is available^59^, and juniper is abundant in preserved woodrat middens across California’s Mojave Desert and Death Valley^60^. The δ^13^C values of RLB coprolites are also broadly consistent with those recovered from tissues of modern *Neotoma fuscipes* populations inhabiting juniper woodlands^61^. Given the exceptional preservation of these coprolites, investigation of additional diet proxies, such as plant cuticles^62^, may be possible.

**Figure 5:**
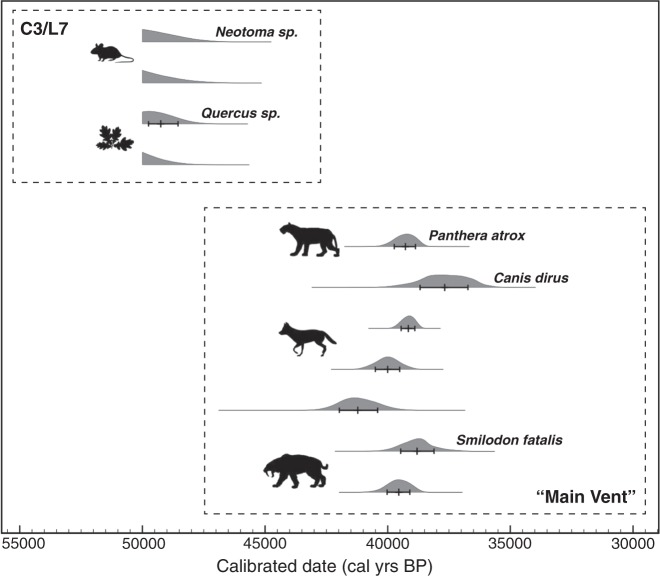
Calibrated radiocarbon dates showing a distinct age grouping of C3/L7 specimens as compared with representative radiocarbon dates of megafauna from the “Main Vent” of Box 1. Dates plotted include the C3/L7 coprolites of *Neotoma* sp. (P23–33721a, P23–33721b) and the oak leaves *Quercus* sp. (P23–39548, P23–39539) associated with them, as reported in this study; *Panthera atrox* (P23–4096), *Canis dirus* (P23–1619, P23–2294, P23–15023, P23–11369), and *Smilodon fatalis* (P23–967, P23–15065) from Fuller *et al*.^21^; an additional 20+ radiocarbon dates from Fuller *et al*. (2019) are consistent with this pattern but not shown for brevity. Figure produced using OxCal v4.3.2 and the Intcal13 calibration curve^26^. The probability distributions for the 4 dates from the present study are truncated because they all extend beyond the end of the IntCal13 curve. Plant and animal symbols modified from Phylopic.

The coprolites differ from the coarser “main vent” of Box 1 in their sedimentary context, the higher asphalt content of their surrounding matrix, and their significantly older age. For example, radiocarbon dates on saber-tooth cat (*Smilodon fatalis*), dire wolf (*Canis dirus*), and American lion (*Panthera atrox*) span ~33–37,000 ^14^C years before present (uncalibrated), as compared with our dates of 46–49,000 ^14^C years before present (uncalibrated)^21,22^ (Fig. 5). This difference is most consistent with a scenario in which there were either two activity periods of the same seep or two different, temporally segregated seeps. While it is possible that the coprolites and associated plant material were buried and then secondarily preserved *in-situ* by the same asphalt seep that generated the “main vent” deposit 10,000 years later, this is unlikely because the very climatic and elevational qualities that have heretofore prevented midden recovery from coastlines would very likely have precluded the exceptional preservation of material seen here for 10,000 years prior to impregnation with asphalt. Combined with a previous insect radiocarbon study at Rancho La Brea^15^, these coprolites suggest that the processes dictating preservation of small organisms such as rodents and plants may differ from those producing non-stratified megafaunal assemblages^15^.

There are three other known fossiliferous asphaltic deposits within California linked to hydrocarbons of the Monterey Formation and their equivalents, all of which were historically excavated in the early-mid 1900s: Carpinteria on the Pacific Coast, and McKittrick and Maricopa inland within the central valley^18^. All three have yielded plant, insect, and vertebrate remains, though they vary in their preservational quality^63^. *Neotoma* skeletal remains have been reported from Carpinteria^64^ and McKittrick’s Sternberg pit (UCMP locality −7139; listed as *N. lepida* and *N. fuscipes*). With renewed study, it is possible that coprolites may be encountered at these localities as well.

Globally, while the genus *Neotoma* is not present in South America, several families of rodents including chinchilla rats (Abrocomidae), viscachas (Chinchillidae), leaf-eared mice (Cricetidae), and degus (Octodontidae) have been used in paleoenvironmental reconstructions because their similarly urine-hardened deposits contain plant macrofossils and pollen^65^. Perhaps future studies of South American asphaltic deposits^23^, with renewed attention to sediment and microfossil excavation protocols, could also yield rodent coprolites and nests of paleoecological relevance. Indeed, a recent study from Tanque Loma in Ecuador hypothesized that plant material associated with a mass death assemblage of ground sloths (*Eremotherium*) could represent disaggregated fecal material^66^. Similarly, the presence of putative “cigar-shaped” coprolites “the size and shape of rodentia” has been reported from the asphaltic site Las Breas de San Felipe of Cuba^67^. In both cases, these promising observations have yet to be tested with quantitative, stratigraphic, and chemical analyses and, in light of our study, warrant further investigation.

Our multidisciplinary study highlights the evolving proxies and techniques that can be employed to reconstruct paleoenvironments at asphaltic localities. We also emphasize the value of paleomitigation endeavors in urban areas, such as Project 23, which can yield unanticipated and high-quality datasets that benefit research questions. Traditional studies at RLB have focused on reconstructing the interactions between megafaunal mammal carnivores and herbivores, e.g., saber-tooth cats and bison^13^ and attempting to track how carnivores respond to climatic and biotic perturbations^14^. However, such studies have yet to incorporate values of primary producers in assessing isotopic change through time, and thus do not account for potential baseline shifts in stable isotopes of nitrogen that may obscure true inferences of trophic position shifts^68^. Prior studies of RLB plant material have been hampered by a lack of stratigraphic control for plant assemblages, and the plant macrofossils in the midden of C3/L7, and potentially others, represent stratigraphically intact material that could be assessed as a coherent assemblage. While *Neotoma* is no longer present in the immediate RLB vicinity of Hancock Park because of extreme habitat modification and isolation from natural areas, the genus is still found in nearby urban-suburban green spaces such as Griffith Park (~9 km away), where sightings of both the rodents and their middens are abundantly recorded on citizen science platforms such as iNaturalist (https://www.inaturalist.org). Thus, the coprolites featured here provide an unexpected baseline of the past, and one that could be connected to the present through new studies of “urban” Los Angeles middens, yielding a transect through time of changing climates, ecosystem baselines, and animal behaviors.

## Methods

Excavation of Rancho La Brea’s Project 23 began in August 2008 as a salvage/mitigation project. Sixteen entire asphaltic deposits were removed from their *in situ* position in one or multiple intact sections. Custom-sized wooden boxes were built around blocks of sediment containing asphaltic deposits and filled in with polyurethane foam and fill dirt to preserve integrity during storage. Boxes range in weight from ~123,000 lbs (Box 1) to ~9,000 lbs (Box 10B). Excavation took place within the “boxes” (Fig. 1C), with walls removed as needed to allow excavation to proceed following standard protocols developed for RLB deposit Pit 91^69^. All grids are oriented with respect to the deposit’s original north orientation, with alphanumeric grid/level combinations excavated in 25 cm spits. Sediments and fossiliferous matrix were placed into 5-gallon metal buckets corresponding to specific grid/level excavation units. Asphalt was removed from the microfossil matrix using heated biodiesel and n-propyl bromide^24^. Cleaned matrix was screened through a #20 ASTM standard mesh (0.85 mm sieve size) initially; such a mesh size is the typical tool for processing *Neotoma* fossil middens^70^. The coprolites were encountered in unit C3/L7 of Box 1 during processing.

We processed additional 2 kg batches of previously unprocessed sediment archived at the La Brea Tar Pits & Museum long-term storage for Project 23 salvage, following a modified version of the museum’s standard protocol^24^ in which sieves of additional size were used (10 meshes ranging from 5/16” to #400 ASTM size) to account for smaller sediments and microfossils. Percent asphalt content was calculated by subtracting the dried, processed sediment weight from its original 2 kg weight. Though we employed fine mesh sizes to capture fine sands and silts, loss during sieving and drying is possible and thus we may slightly overestimate asphalt content due to sediment weight loss through processing. Unfortunately, initial salvage excavations did not retain bulk unprocessed sediment for the “main vent” A1–2/B1–2; we therefore used material from highly similar deposits from Project 23’s Box 13, B3/L5 grid for direct quantitative comparison^25^.

We produced radiocarbon dates for two individual coprolites (P23–33721 batch catalogued) and two sets of directly associated oak leaves (P23–39548, P23–39539), thus accounting for potential differences in age of deposition. While asphalt is an effective preservative agent of organic material, it is also a hydrocarbon contaminant that will skew dates to artificially older ages. Residual asphalt was removed using toluene/methanol rinses until liquid was clear, followed by methanol and water^21^. We performed an acid-base-acid wash (1 N HCL and 1 N NaOH at 75 °C) and bleached samples to holocellulose (1:1 mixture of 1 N HCl and 1 M NaClO_2_) prior to combustion. Graphitized specimens were run at UC Irvine W.M. Keck Carbon Cycle Accelerator Mass Spectrometer Facility. Using OxCal v.4.3.2, we found the probability distributions of the resulting dates to be outside the range of the calibration curve IntCal13 and could not confidently report a calibrated median or 95.4% age range^26^. Following the above pre-treatment procedures for radiocarbon dating, we produced stable isotope values of δ^13^C only; bleaching precludes analysis of δ^15^N. δ^13^C values were measured to a precision of <0.1‰ using a Fisons NA1500NC elemental analyzer/Finnigan Delta Plus isotope ratio mass spectrometer. We present %C as the carbon percentage in the treated cellulosic material measured.

We generated high-resolution SEM images of four individual coprolites that were cleaned following standard biodiesel/n-propyl bromide protocols, with two imaged whole (P23–33820, P23–33821) and two splits (P23–38822, P23–38819). We sputter-coated individual coprolites in a mixture of gold and palladium using an Emitech K550x sputter coater to provide conductive metal coating for SEM. Images were generated using a Hitachi S-3000N variable-pressure scanning electron microscope at the Natural History Museum of Los Angeles County.

We measured the length and width of 431 fecal pellets from Box 1’s unit C3/L7 using digital calipers to the nearest 0.1 mm. Following standard protocol^29^, we dropped the 50% narrowest pellets by width to avoid inclusion of subadults. We also present the mean of the widest top 50, top 20, and top 10 by width, as it has been shown to be less sensitive to sampling method^27,29^.We assume that these fecal pellets represent multiple generations of individuals and therefore are a population-level estimate, rather than the deposition of an individual rodent, as is known from modern woodrat midden studies^27^. We estimated body size using an equation^29^ (y = 0.005×+ 3.559, r^2^ = 0.69, p < 0.0001) that relates body mass to width of the fecal pellets. For a comparative reference, we collected values on modern fecal width from the literature for *Neotoma fuscipes*^35^ and *Neotoma lepida*^71^ – two species that co-occur in the Los Angeles county area based on specimen occurrences and their associated metadata at the Natural History Museum of Los Angeles County. We generated a comparative reference set of body masses for these two species from weight recorded at capture for *Neotoma macrotis* (historically *Neotoma fuscipes macrotis*^31^) (N = 30) and *Neotoma lepida* (N = 59) caught in Los Angeles county. While we use the species names *fuscipes* and *lepida* to remain consistent with local museum collections, we recognize there have been recent systematic revisions for the *Neotoma lepida* group^30^, and *Neotoma fuscipes macrotis* has been elevated to *Neotoma macrotis*^31^; we are primarily interested in an identification at the genus level.

## Data accessibility

All fossil specimens are publicly available at the La Brea Tar Pits & Museum collections and comparative modern specimens are publicly available at the LACM Mammalogy collection. All relevant data are in the manuscript or are available as supplemental materials.

## Supplementary information

Supplementary information.
